# Massachusetts Cattle Commission

**Published:** 1896-10

**Authors:** 


MASSACHUSETTS CATTLE COMMISSION. As we go to press the news reaches us of the failure of the Bay State Governor to reappoint on the Commission our esteemed colleague, Dr. C. P. Lyman. At first we were inclined to believe that this action was the outcome of failure on the part of the Governor to appreciate the valuable services rendered the people of that Commonwealth by Dr. Lyman during his long years of unselfish labor. But we are glad to learn that the Governor's decision carries with it no reflection upon Dr. Lyman's worth as a well-equipped member of the Commission; no criticism of his high and unselfish interest in behalf of his fellow-citizens, and no failure to appreciate the high standard of scientific light that he has ever striven to bring out for the guidance of this Commission, through all its varied and valuable work, in the many years of studious toil in working out the great sanitary problems that have vexed and perplexed the scientific world as to the best and most effective methods for dealing effectually with these momentous questions. Such a man can never lose the esteem and admiration of an intelligent people, who will always appreciate the great benefit they have derived from his unselfish efforts in behalf of his fellow-men.

---

DR. JOHN M. PARKER, of Haverhill, Mass., who succeeds Prof. Lyman, has given the subject of tuberculosis much consideration and study, and will bring to the Commission a knowledge of this disease that should be invaluable when directed along the lines now so generally accepted as the only true plan for the better control and ultimate eradication of this great scourge. The responsibilities that will directly fall upon his shoulders in the sharing of the work of the Commission, will, we are sure, be keenly appreciated by him, and the force of their importance be thoroughly measured. We congratulate Dr. Parker on his appointment, and the best wishes that we can extend to him are that he may win for himself in this new field of responsibility the same laurels of esteem, appreciation, and admiration which have been so generously conceded his predecessor by the members of our profession, whose ability to judge of the merits of such public service is acknowledged by all.

---

Our November number will be an unusually attractive issue. In addition to its regular compilations of what the veterinary world is doing—our Societies, the profession, and abstracts of the best thoughts of the brainiest men of the world—it will contain the opening address to the Harvard Veterinary students by Prof. Whitney, a most thoughtful presentation to young men entering upon a course of scientific study; a paper on Parturient Apoplexy by one of Pennsylvania's most practical veterinarians; an article on Canine Distemper by one who has given this important subject many years of careful study under special opportunities; a review of the question of Homœopathy in Veterinary Science as compared with the merits of the regular school, by one who gave several years of patient study to equip himself to practise this branch of the healing art, and who is recognized among the practitioners of his State as a thorough student, a well-read practitioner, and one of the most thoughtful devotees of the profession. In addition to these papers there will be thirty-five to forty pages of the proceedings of the thirty-third annual convention of the U. S. V. M. A.

---

The Journal has just closed a five years' contract with the Publication Committee of the United States Veterinary Medical Association to publish each year the entire proceedings of the annual convention, and we have decided to give our readers the benefit of this valuable acquisition of reading matter, which must be of great interest to the veterinary profession. Each issue of the Journal will contain from thirty-five to forty pages of this matter, and will be so arranged that it may be readily removed from the Journal and bound separately. This will be given to our readers at no additional cost, and the other departments of the JOURNAL that have made it of so much interest to our profession will be retained, with the addition from time to time of everything new and useful to our brother veterinarians.

---

OBSERVANCE of the code of ethics among English veterinarians has become a serious matter of controversy and is provoking a conflict of opinions. The many objectionable forms of advertising, the unbusiness-like methods, have been too long tolerated among English veterinarians to be easily eliminated and corrected at this late day.

---

IMPORTANT NOTICE. The fall examination of the Pennsylvania State Board of Veterinary Medical Examiners will take place at Harrisburg on the 20th and 21st days of October, at 10 A.M. each day. Those desiring to present themselves will at once file their applications with the Secretary, Dr. S J. J. Harger, 205 North Twentieth Street, Philadelphia, Pa. By order of the President, W. Horace Hoskins.

